# Three new species of the genus *Chilocorellus* Miyatake (Coleoptera, Coccinellidae, Sticholotidini) from the Philippines

**DOI:** 10.3897/zookeys.937.50139

**Published:** 2020-06-01

**Authors:** Xiaoning Zhang, Xinyue Liang, Xiaosheng Chen, Xingmin Wang

**Affiliations:** 1 Key Laboratory of Bio-Pesticide Innovation and Application, Engineering Technology Research Center of Agricultural Pest Biocontrol, Ministry of Education & Guangdong Province; Department of Entomology, South China Agricultural University, Guangzhou 510640, China South China Agricultural University Guangzhou China; 2 Department of Forest Protection, College of Forestry and Landscape Architecture, South China Agricultural University, Guangzhou, 510640, China South China Agricultural University Guangzhou China

**Keywords:** *
Chilocorellus
*, Coccinellidae, Coccinelloidea, Coleoptera, new species, Philippines

## Abstract

The genus *Chilocorellus* Miyatake, 1994 has been revised. Three new species (*C. uncinacanthus* Zhang & Wang, **sp. nov.**, *C. denspinulifer* Zhang & Wang, **sp. nov.**, and *C. fistulachaetodontus* Zhang & Wang, **sp. nov.**) from the Philippines are described and illustrated in the present paper. An updated key to the species of the genus *Chilocorellus* is provided. In addition, a list of all known species and their distributions is also provided.

## Introduction

The subfamily Sticholotidinae was recognized by Weise (1887) in the modern sense as a peculiar group of the family Coccinellidae. It has been used to accommodate small or very small ladybird beetles in which the form of the terminal maxillary palpomere is not securiform and is elongate conical or apically acuminate (Gordon 1977, Ślipiński 2004). Gordon (1977) and Hoàng (1982) used Sticholotidini after “Sticholotini” was first-used by Weise (Weise 1887, 1901). Miyatake (1994) revised the subfamily Sticholotidinae from Asia and established six new genera (*Synonychimorpha*, *Chilocorellus*, *Sulcolotis*, *Filipinolotis*, *Mimoserangium*, and *Coelolotis*) in the tribe Sticholotidini.

*Chilocorellus* Miyatake, 1994 was described from Luzon, Philippines with *C. luzonicus* as the type species. Subsequently three new species (*C. quadrimaculatus*, *C. protuberans*, and *C. tenuous*) from China were described by Wang and Ren (2010). In 2011, another species (*C. seleuyensis*) from Laos was added to this genus by Wang and Ren. The molecular phylogenetic analyses revealed no significant support for the tribe Sticholotidini (Giorgi et al. 2009; Magro et al. 2010; Seago et al. 2011; Robertson et al. 2015) and Sticholotidini was placed into an expanded concept of Coccinellinae (Escalona and Ślipiński 2012; Ślipiński 2007). The taxonomic status of the genus *Chilocorellus* has changed. Seago et al. (2011) investigated the phylogeny and evolution of the Coccinellidae based on the combination of molecular and morphological data. Their results showed that *Chilocorellus* was embedded in the tribe Chilocorini and recovered as the sister group of *Chilocorus*. They transferred *Chilocorellus* from Sticholotidini to Chilocorini. Recently, Li et al. (2020) reconstructed the phylogeny of the tribe Chilocorini. The results indicated that the two unidentified specimens of *Chilocorellus* were forming a single branch. However, they excluded *Chilocorellus* from Chilocorini as it was recovered far from this tribe based on combined molecular and morphological data analyses.

In this study, examination of ladybird specimens from the Australian National Insect Collection revealed that three species belong to this genus and they are described herein as new to science.

## Materials and methods

The specimens of the new species were collected from Luzon, Philippines. All examined materials are preserved in the Australian National Insect Collection, CSIRO, Canberra, Australia (**ANIC**) and the Insect Collection of South China Agricultural University, Guangzhou, China (**SCAU**). External morphology was observed with a dissecting stereoscope (Zeiss Discovery. V20). The following measurements were made with an ocular micrometer following Wang et al. (2017): total length (**TL**), length from apical margin of clypeus to apex of elytra; total width (**TW = EW**), width across both elytra at widest part; height (**TH**), from the highest part of the beetle to elytral outer margins; head width (**HW**) in a frontal view, widest part including eyes; pronotal length (**PL**), from the middle of anterior margin to the base of pronotum; pronotal width (**PW**) at widest part; elytral length (**EL**), along the suture, from the apex to the base including the scutellar shield; eyes width (**Eye W**) in a frontal view.

Images were taken with digital cameras (ZEISS Imager M2 and Axiocam 506 Color) connected to a dissecting microscope. The software ZEN 2.3 was used to capture images from the cameras. And Adobe Photoshop CC was used to clean up images. The distribution map was downloaded from a free map website (http://alabamamaps.ua.edu).

Terminology follows Ślipiński (2007) and Ślipiński and Tomaszewska (2010). Type specimens designated in the present paper are deposited in ANIC.

## Taxonomy

### Genus Chilocorellus

Taxon classificationAnimaliaColeopteraCoccinellidae

Miyatake, 1994

78EDFD52-141E-5A14-BE89-82EDFE2EC1D6

#### Type species.

*Chilocorellus luzonicus* Miyatake, 1994.

#### Diagnosis.

**Figure 1.**
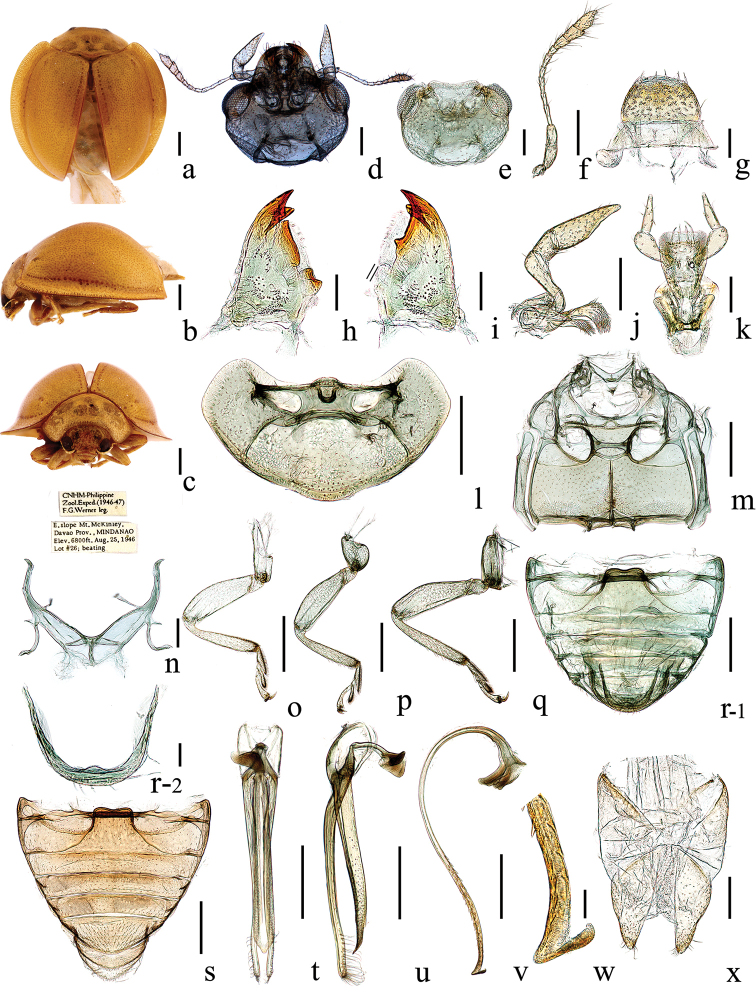
*Chilocorellus uncinacanthus* sp. nov. **a** dorsal habitus **b** lateral habitus **c** frontal habitus **d** head, ventral **e** head, ventral **f** antenna **g** labrum **h** left mandible **i** right mandible **j** maxilla **k** labium **l** prothorax, ventral **m** mesoventrite and metaventrite **n** metendosternite **o** front leg **p** mid leg **q** hind leg **r-1** male abdomen **r-2** male terminalia **s** female abdomen **t** tegmen, ventral view **u** tegmen, lateral view **v** penis, lateral **w** apex of penis **x** female genitalia (ovipositor). Scale bars: 0.5 mm (**a–c, l–m, o–v**) ; 0.2 mm (**d–f, j, n, x**); 0.1 mm (**g–i, k**); 0.05 mm (**w**).

*Chilocorellus* is similar to *Synonychimorpha* Miyatake, 1994 in general appearance, with body rounded and glabrous; dorsal surface predominantly yellowish, elytral epipleuron broad (Fig. 1a–c); antenna with 11-antennomeres, long, antennal club distinctly 3-antennomeres, terminal antennomere elongate, and apically distinctly pointed (Fig. 1d, f). It can be distinguished from *Synonychimorpha* by its prosternal process in the form of an approximately ovoid prominence without carinae (Fig. 1l). In *Synonychimorpha*, prosternal process is square.

#### Description.

Body rounded, moderately to strongly convex, sub-hemispherical; dorsal surface apparently glabrous (Fig. 1a–c).

**Figure 2.**
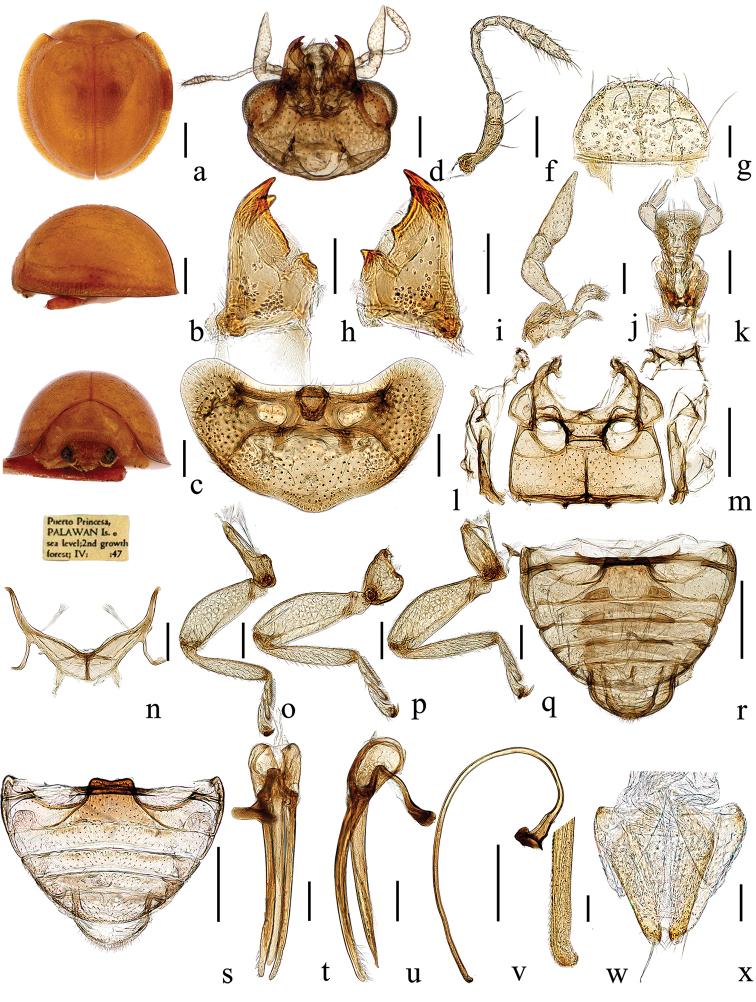
*Chilocorellus denspinulifer* sp. nov. **a** dorsal habitus **b** lateral habitus **c** frontal habitus **d** head, ventral **f** antenna **g** labrum **h** left mandible **i** right mandible **j** maxilla **k** labium **l** prothorax, ventral **m** mesoventrite and metaventrite **n** metendosternite **o** front leg **p** mid leg **q** hind leg **r** male abdomen **s** female abdomen **t** tegmen, ventral view **u** tegmen, lateral view **v** penis, lateral **w** apex of penis **x** female genitalia (ovipositor). Scale bars: 0.5 mm (**a–c, m, r–s, v)**; 0.2 mm (**d, l, n–q, t–u; f**), 0.1 mm (**h–k, x**); 0.05 mm (**g, w**).

**Figure 3.**
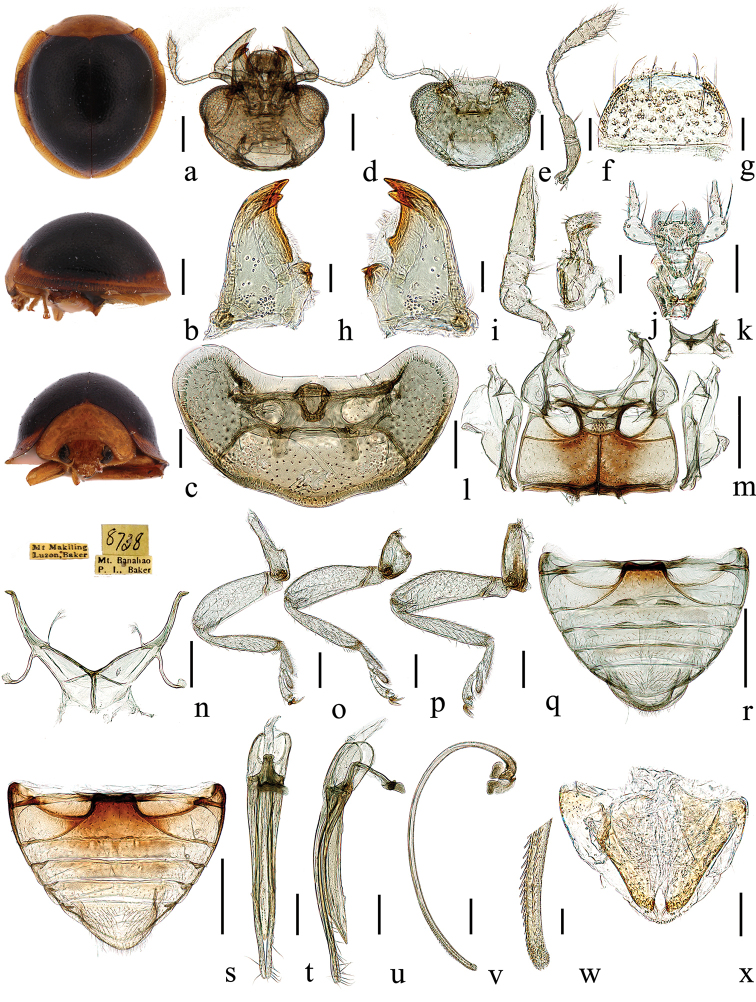
*Chilocorellus fistulachaetodontus* sp. nov. **a** dorsal habitus **b** lateral habitus **c** frontal habitus **d** head, ventral **e** head, ventral **f** antenna **g** labrum **h** left mandible **i** right mandible **j** maxilla **k** labium **l** prothorax, ventral **m** mesoventrite and metaventrite **n** metendosternite **o** front leg **p** mid leg **q** hind leg **r** male abdomen **s** female abdomen **t** tegmen, ventral view **u** tegmen, lateral view **v** penis, lateral **w** apex of penis **x** female genitalia (ovipositor). Scale bars: 0.5 mm (**a–c, m, r–s**); 0.2 mm (**d–e, l, n–q, t–v**); 0.1 mm (**f, j–k, x**); 0.05 mm (**g, h–i, w**).

Head strongly hypognathous and small (Fig. 1c, d). Frons wide and flat with punctate (Fig. 1d). Clypeus short and transverse with anterior margin emarginate medially (Fig. 1e). Eyes large, coarsely faceted, inner eye canthus absent (Fig. 1c, d). Antennae with eleven antennomeres, scape and pedicel robust, scape elongate and curved near base, pedicel approximately as broad as scape; flagellum with nine antennomeres, antennomeres 3–5 slender, antennomeres 6–8 subequal in length and width; three terminal antennomeres comparatively wider than other antennomeres, forming a fusiform club with setae (Fig. 1d, f). Labrum transverse, rounded anteriorly and covered with long dense setae (Fig. 1g). Mandible subtriangular with two apical teeth, sharp and smooth; the two parts of mandibles asymmetrical, mola normal with two molar teeth on the left and one molar tooth on the right (Fig. 1h, i). Maxillary palp with four palpomeres, palpomere 1 small, palpomere 2 geniculate and at least two times as long as wide, palpomere 3 obviously short, terminal one slender and sharp, with strong obliquely truncated apex (Fig. 1j). Labial palp with three palpomeres, palpomere 1 tiny, palpomere 2 robust, terminal palpomere slender with setae, strongly conical, pointed apically; ligula membranous; insertion of labial palps visible ventrally on prementum; mentum trapezoidal, distinctly narrowed basally (Fig. 1k). Pronotum moderately transverse, broadly arcuate in both of frontal and lateral view, anterior margin emarginate; hind margin rounded; lateral margin rounded; not conspicuously angulate (Fig. 1a–c). Scutellar shield triangular (Fig. 1a, c). Elytra with prominent humeral angles, convex, anterior margin almost equal in width to hind margin of pronotum, lateral margins rounded, broadly explanate; dorsal surface glabrous, with dense and similar-sized punctation (Fig. 1a–c). Elytral epipleuron broad and gently complete apically, approximately 1/4 width of elytra. Hind wings well developed (Fig. 1a, b). Prosternum T-shaped, with golden pubescence and inconspicuous punctures; prosternal process significantly narrower than the transverse procoxa, prosternal carina absent; hypomeron broad without foveate (Fig. 1l). Meso- and metaventral processes broad, as wide as midcoxal diameter, with golden pubescence and inconspicuous punctures; metaventral postcoxal lines angled at the middle joint and complete (Fig. 1m). Metendosternite stalk distinctly shorter (0.5 or less) than broad, tendons widely separated and placed near apices of arms (Figs 1n, 2n, 3n). Legs robust with dense pubescence; pro and hind coxae transverse but mid coxae oval; trochanter sub-triangular, robust; femora thick, as long as tibia, but tibia slender, half as wide as femora; tarsi with four tarsomeres, tarsomere 3 minute, tarsomere 4 slender, longer than other tarsomeres; claws bidentate with two teeth (Fig. 1o–q). Abdomen with five ventrites; ventrite 1 slightly longer than ventrite 2, abdominal postcoxal lines incomplete, not recurved reaching the hind margin of ventrite 1; ventrites 2–4 sub-equal in length; ventrite 5 longer than ventrite 4, with hind margin rounded (Fig. 1r–1, s). Segment VIII, hind margin of male sternite emarginate and hind margin of female sternite rounded; tergite with hind margin rounded (Fig. 1r–1, s). Male terminalia, sternite IX and X sclerotized, with hind margin rounded (Fig. 1r–2). Male genitalia: tegmen slender and symmetrical, basal piece membranous; tegminal strut T-shaped and widened apically; penis guide slender in ventral and lateral views, parameres slender, setose at apex. Penis slender and long, curved; penis capsule asymmetrical, inner arm developed, outer arm reduced; the front part and apex of penis tubular with teeth (Fig. 1t, u). Female genitalia: coxites triangular, setose apically; styli conspicuous (Fig. 1x).

#### Distribution.

**Figure 4.**
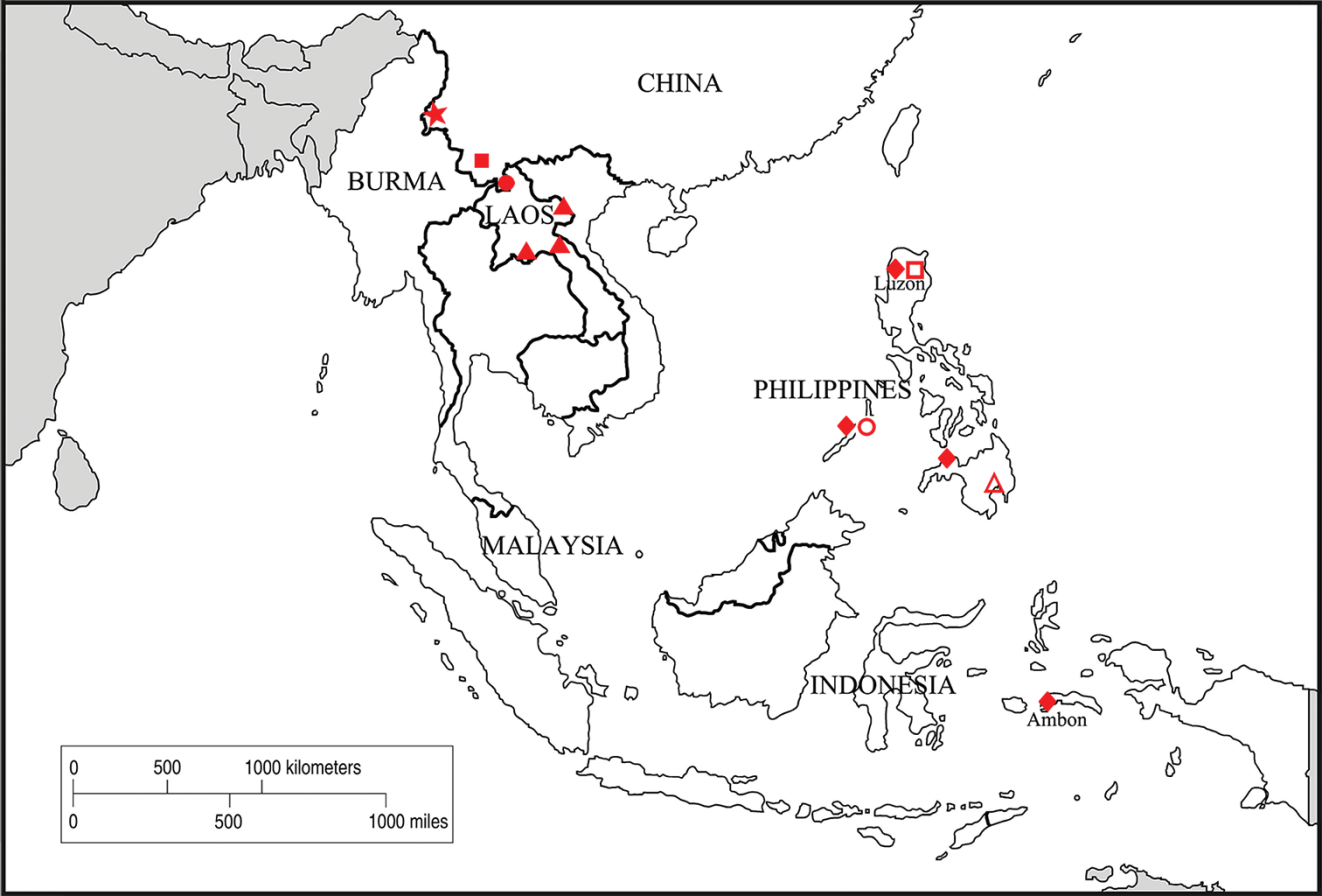
Distribution map. *C. luzonicus* Miyatake, 1994 (♦); *C. quadrimaculatus* Wang & Ren, 2010 (★); *C. protuberans* Wang & Ren, 2010 (●); *C. tenuous* Wang & Ren, 2010 (■); *C. seleuyensis* Wang & Ren, 2011 (▲); *C. uncinacanthus* Zhang et Wang, sp. nov. (▲); *C. denspinulifer* Zhang et Wang, sp. nov. (●); *C. fistulachaetodontus* Zhang et Wang, sp. nov. (■).

China, Indonesia, Laos, Philippines (Fig. 4).

### Chilocorellus uncinacanthus

Taxon classificationAnimaliaColeopteraCoccinellidae

Zhang & Wang sp. nov.

3258409F-257C-55C6-9487-A7985C293A3D

http://zoobank.org/7D8DFD2B-33EC-4F08-85FF-B42D484E628C

Figures 1 , 4

#### Holotype.

Philippines: 1 male, CNHM Philippines Zool. Exped. (1946–47) F. G. Werner leg., E. slope Mt. Mckinley, Davao Province, MINDANAO Elev. 6800 ft., 25 Aug. 1946 Lot #26, beating.

#### Paratypes.

1 female, CNHM Philippines Zool. Exped. (1946–47) H. Hoogstraal leg.; Lake Linau, E. slope Mt. Apo, Davao Province, MINDANAO Elev. 7900 ft., mossy forest. 2 Nov. 1946. 1 female, CNHM Philippines Zool. Exped. (1946–47) H. Hoogstraal leg.; Baclayan, E. slope of Mt. Apo, Davao Province., MINDANAO Elev. 6500 ft., original forest. Nov. 1946. 1 female, CNHM Philippines Zool. Exped. (1946–47) H. Hoogstraal leg.; Lake Linau, E. slope Mt. Mckinley, Davao Province, MINDANAO Elev. 7900 ft. stunted mossy forest, 11 Jun. 1946.

#### Diagnosis.

This species is similar to *C. ﻿protuberans*, *C. ﻿tenuous*, and *C. seleuyensis* in general appearance (e.g., the elytra yellow without any spots and broad), but can be distinguished from them by the anterior part and apex of penis hatchet-shaped with irregularly serrated coupled teeth. In *C. protuberans*﻿, the penis is long and slender, with a large penis capsule and apex of penis is curved, with many small teeth. In *C. ﻿tenuous*﻿, penis is very long and slender and apex of penis has many large teeth. In *C. ﻿seleuyensis*, penis is longer than in other species and apex of penis is partly membranous, with many small teeth.

#### Description.

TL: 3.26–3.30 mm, TW: 2.87–3.02 mm, TH: 1.29–1.40 mm, TL/TW: 1.09–1.14; PL/PW: 0.23–0.24; EL/EW: 0.86–0.98 HW/PW: 0.55–0.58; PW/TW: 0.54–0.55; HW/TW: 0.30–0.32; Eye W/HW: 0.5–0.57.

Head yellow, with eyes silvery gray. Pronotum, scutellar shield, and elytra yellow, with small dense punctures. Underside yellow, except mesoventrite and metaventrite yellowish brown.

Body oval, moderately convex (Fig. 1b, c). Head small, 0.3 times elytral width (HW/TW = 1:3.2) with sparse pubescence. Eyes oval, widest interocular distance 0.54 times head width (eye W/HW = 1:1.87). Frons broad with irregular transparent spots, punctures uniform and dense (Fig. 1c, d).

Pronotum 0.55 times elytral width (PW/TW = 1: 1.83), moderately transverse, with irregular transparent spots, punctures uniform and dense (Fig. 1c). Elytra with transparent humeral angles, punctures uniform and dense (Fig. 1a–c). Male genitalia (Fig. 1t–w): penis guide in lateral view wide at base and uniformly narrowing to pointed apex; parameres distinctly longer than penis guide, uniformly slender with densely distributed long setae apically (Fig. 1t, u); penis tubular, extremely long, curved; flabellate part of penis capsule very broad, anterior part and apex of penis hatchet-shaped with irregularly serrated coupled teeth (Fig. 1v, w).

#### Distribution.

Philippines (Davao).

#### Etymology.

The name *uncinacanthus* is composed of the Latin word *uncin*, which refers to the anterior part of uncinate penis and *acantha*, referring to the anterior part and apex of the penis.

### Chilocorellus denspinulifer

Taxon classificationAnimaliaColeopteraCoccinellidae

Zhang & Wang sp. nov.

19468C72-39D1-5B95-B717-E01AB818E917

http://zoobank.org/8505FE62-5070-44FE-BA1A-1CD6EFE1E8B8

Figures 2 , 4

#### Holotype.

Philippines: 1 male, Puerto Princesa, Palawan Is, sea level, secondary growth forest, IV 47.

#### Paratype.

1 female, Philippines, Puerto Princesa, Palawan Baker.

#### Diagnosis.

This species is similar to *C. uncinacanthus*﻿, *C. protuberans*﻿, *C. ﻿tenuous*, and *C. ﻿seleuyensis*﻿ by the strongly convex, yellow elytra having no spots. But unlike these species, its body is small, the anterior and apex of the penis is tubular with irregular dense tiny teeth. In *C. uncinacanthus*, the apex of the penis is hatchet-shaped and bears large teeth; in *C. protuberans*, the apex of the penis is curved and membranous, with many small teeth; in *C. ﻿tenuous*, the apex of the penis is straight and membranous, with many asymmetrical large teeth.

#### Description.

TL: 2.33–2.40 mm, TW: 2.26–2.28 mm, TH: 1.11–1.21 mm, TL/TW: 1.03–1.05; PL/PW: 0.37–0.47; EL/EW: 0.94–0.97 HW/PW: 0.53–0.55; PW/TW: 0.58–0.59; HW/TW: 0.31–0.32; Eye W/HW: 0.33–0.41.

Head yellow, with eyes silver-gray. Pronotum, scutellar shield, and elytra uniformly yellow, with tiny dense punctures. Underside yellow; prosternum, mesoventrite, metaventrite, and legs yellowish brown.

Body rounded, strongly convex (Fig. 2b, c). Head small, 0.32 times elytral width (HW/TW = 1:3.2), with sparse pubescence. Eyes oval, widest interocular distance 0.37 times head width (eye W/HW = 1:2.7). Frons broad, punctures uniform and dense (Fig. 2c, d).

Pronotum 0.59 times elytral width (PW/TW = 1:1.7), moderately transverse, punctures uniform (Fig. 2a, c). Elytra with humeral angles, punctures uniform and dense (Fig. 2a–c). Male genitalia (Fig. 2t–w): penis guide wide at base in lateral view and uniformly narrowing to pointed apex; parameres obviously longer than penis guide, uniformly slender with densely distributed long setae apically (Fig. 2t, u); penis tubular, extremely long, curved; flabellate part of penis capsule broad, anterior and apex of penis with irregular tiny dense teeth, and apex of penis nest-shaped (Fig. 2v, w).

#### Distribution.

Philippines (Puerto Princesa).

#### Etymology.

The name *denspinulifer* is composed of the Latin word *dens*, meaning dense, and *spinulifer*, which refers to the part of the penis with spinulose appendage.

### Chilocorellus fistulachaetodontus

Taxon classificationAnimaliaColeopteraCoccinellidae

Zhang & Wang sp. nov.

0CF9D675-FA45-5CC6-8E95-7A44947FEA00

http://zoobank.org/F57C9A4F-0185-4140-8717-2C13CCAF6163

Figures 3 , 4

#### Holotype.

Philippines: 1 male, Mt Makiling, Luzon, Baker.

#### Paratypes.

1 female, Mt Maklling, Luzon, Baker; 1female, Mt Banahaw, Philippines, Luzon, Baker; 1 female, Philippines, Luzon: Lagunas Mt Banahaw nr acool ca. 1 km from Kinabuhayan, 500 m, degraded rain forest, 28 Nov. 1998.

#### Diagnosis.

This species can be distinguished from the other species of the *Chilocorellus* by following characters: body is small; elytra are black with just yellow margin; apex of penis guide with a membranous triangular prominence; penis long, anterior part and apex of penis with teeth, and apex of penis simple.

#### Description.

TL: 2.20–2.37 mm, TW: 1.98–2.14 mm, TH: 1.19–1.21 mm, TL/TW: 1.10–1.11; PL/PW: 0.37–0.44; EL/EW: 1.01–1.02; HW/PW: 0.50–0.56; PW/TW: 0.61–0.65; HW/TW: 0.32–0.34; Eye W/HW: 0.55–0.56.

Head yellow, eyes black. Pronotum, scutellar shield, and elytral epipleuron yellow. Elytra black with yellow margin. Underside yellow; prosternum, mesoventrite, metaventrite, and legs dark brown.

Body approximately rounded, strongly convex (Fig. 3a–c). Head small, 0.33 times elytral width (HW/TW = 1:3.0), with sparse pubescence. Eyes oval, widest interocular distance 0.56 times head width (eye W/HW = 1:1.8). Frons broad, punctures uniform and dense (Fig. 3c, d).

Pronotum 0.63 times of elytral width (PW/TW = 1:1.6), moderately transverse, punctures uniform (Fig. 3a, c). Elytra with black humeral angles, punctures uniform and dense (Fig. 3a–c). Male genitalia (Fig. 3t–w): penis guide wide, uniformly narrowing to pointed apex, apex of penis guide with a membranous triangular prominence. Parameres extremely slender, longer than penis guide, uniformly slender with densely distributed long setae apically (Fig. 3t, u). Penis tubular, long, curved; flabellate part of penis capsule broad, anterior part and apex of penis with dense small teeth, and apex of penis simple (Fig. 3v, w).

#### Distribution.

Philippines (Luzon).

#### Etymology.

The name *fistulachaetodontus* is composed of the word *fistula*, which refers to the penis shape, and *chaetodontus*, which refers to the anterior part and apex of penis with irregular short, dense teeth.

### An updated key to the species of *Chilocorellus* Miyatake

**Table:** 

1	Dorsal surface bicolored, pronotum and scutellar shield all yellow, but elytra yellow with dark spots or elytra black with yellow margin	**2**
–	Dorsal surface uniformly yellow, without spots	**4**
2	Elytra yellow with dark spots	**3**
–	Elytra black with yellow margin; anterior part and apex of penis with irregular short, dense teeth, and apex of penis siphon-shaped. Distributed in Philippines	***fistulachaetodontus* Zhang et Wang, sp. nov.**
3	A longitudinal oval spot on elytral suture. Distributed in the Philippines	***luzonicus* Miyatake**
–	Four large round spots on elytra, apex of penis with many oppositely arranged large teeth; parameres slightly longer than penis guide; apex of penis pointed and curved. Distributed in China	***quadrimaculatus* Wang & Ren**
4	Parameres prominently longer than penis guide	**5**
–	Parameres slender, slightly longer than or as long as penis guide, apex of penis with many large teeth. Distributed in China	***tenuous* Wang & Ren**
5	Penis tubular, long and simple curved	**6**
–	Penis extremely long, strongly curved; apex of penis partly membranous, with dense small teeth. Distributed Laos	***seleuyensis* Wang & Ren**
6	Apex of penis with membranous uncinus or hatchet-shaped	**7**
–	Apex of penis nest-shaped, with serrated appendage. Distributed in Philippines	***denspinulifer* Zhang et Wang, sp. nov.**
7	Apex of penis with membranous uncinus, with many small teeth. Distributed in China	***protuberans* Wang & Ren**
–	Apex of penis hatchet-shaped, with many small teeth. Distributed in the Philippines	***uncinacanthus* Zhang et Wang, sp. nov.**

## List of species of *Chilocorellus* Miyatake, 1994

***Chilocorellus luzonicus* Miyatake, 1994: 249**

**Distribution.** Philippines (Luzon, Mindanao, Palawan); Indonesia (Ambon).

***Chilocorellus quadrimaculatus* Wang & Ren, 2010: 205.**

**Distribution.** China (Yunnan).

***Chilocorellus protuberans* Wang & Ren, 2010: 205.**

**Distribution.** China (Yunnan).

***Chilocorellus tenuous* Wang & Ren, 2010: 208.**

**Distribution.** China (Yunnan).

***Chilocorellus seleuyensis* Wang & Ren, 2011: 123.**

**Distribution.** Laos (Xam Nua, Vientiane, Bolikhamxai).

***Chilocorellus uncinacanthus* sp. nov.**

**Distribution.** Philippines (Davao).

***Chilocorellus denspinulifer* sp. nov.**

**Distribution.** Philippines (Puerto Princesa).

***Chilocorellus fistulachaetodontus* sp. nov.**

**Distribution.** Philippines (Luzon).

## Supplementary Material

XML Treatment for Chilocorellus

XML Treatment for Chilocorellus uncinacanthus

XML Treatment for Chilocorellus denspinulifer

XML Treatment for Chilocorellus fistulachaetodontus

## References

[B1] Escalona HE Ślipiński SA (2012) Generic revision and phylogeny of Microweiseinae (Coleoptera: Coccinellidae). Systematic Entomology 37: 125–171. 10.1111/j.1365-3113.2011.00601.x

[B2] Giorgi JA Vandenberg NJ McHugh JV Forrester JA Ślipiński SA Miller KB Shapiro LR Whiting MF (2009) The evolution of food preferences in Coccinellidae. Biological Control 51: 215–231. 10.1016/j.biocontrol.2009.05.019

[B3] Gordon RD (1977) Classification and phylogeny of the New World Sticholotidinae (Coccinellidae). The Coleopterist Bulletin 31: 185–228.

[B4] Hoàng DN (1982) Ladybird (Coleopetera: Coccinellidae) of Vietnam. Part 1. Nha xuat ban khoa hoc va kythuat, 211 pp.

[B5] Li WJ Łączyński P Escalona HE Eberle J Huo LZ Chen XS Huang WD Chen BX Ahrens D Ślipiński A Tomaszewska W Wang XM (2020) Combined molecular and morphological data provide insights into the evolution and classification of Chilocorini ladybird (Coleoptera: Coccinellidae). Systematic Entomology 45: 447–463. 10.1111/syen.12409

[B6] Miyatake M (1994) Revisional studies on Asian genera of the subfamily Sticholotidinae (Coleoptera: Coccinellidae). Memoirs of the College of Agriculture, Ehime University 38: 254–256.

[B7] Magro A Lecompte E Magne F Hemptinne J Crouau-Roy B (2010) Phylogeny of ladybirds (Coleoptera: Coccinellidae): are the subfamilies monophyletic? Molecular Phylogenetics and Evolution 54: 833–848. 10.1016/j.ympev.2009.10.022 19903531

[B8] Robertson JA Ślipiński SA Moulton M Shockley FW Giorgi A Lord NP McKenna DD Towaszewska W Forrester J Miller KB Whiting MF McHugh JV (2015) Phylogeny and classification of Cucujoidea and the recognition of a new superfamily Coccinelloidea (Coleoptera: Cucujiformia). Systematic Entomology 40(4): 745–778. 10.1111/syen.12138

[B9] Seago AE Giorgi JA Li J Ślipiński SA (2011) Phylogeny, classification and evolution of ladybird beetles (Coleoptera: Coccinellidae) based on simultaneous analysis of molecular and morphological data. Molecular Phylogenetics and Evolution 60(1): 137–151. 10.1016/j.ympev.2011.03.015 21426943

[B10] Ślipiński A (2004) Revision of the Australian Coccinellidae (Coleoptera). Part 2. Tribe Sticholotidini. Annales Zoologici (Warszawa) 54(2): 389–402.

[B11] Ślipiński A (2007) Australian ladybird beetles (Coleoptera: Coccinellidae) their biology and classification. ABRS, Canberra, 286 pp.

[B12] Ślipiński A Tomaszewska W (2010) Coccinellidae Latreille, 1802. In: Leschen RAB Beutel RG Lawrence JF (Eds) Handbook of Zoology, Vol.2, Coleoptera. Walter de Gruyter GmbH & Co. KG, Berlin/New York, 454–472. 10.1515/9783110911213.454

[B13] Weise J (1887) Feststellung der Gattung *Coleopterus* Muls. Deutsche Entomologische Zeitschrift 31: 183–185. 10.1002/mmnd.48018870130

[B14] Weise J (1901) Coccinelliden aus Ceylon gesammelt von Dr. Horn. Deutsche Entomologische Zeitschrift 44(1901): 417–448. 10.1002/mmnd.48019000237

[B15] Wang XM Ren SX (2010) The genus *Chilocorellus* Miyatake (Coleoptera: Coccinellidae) from China. Annales Zoologici 60(2): 203–208. 10.3161/000345410X516858

[B16] Wang XM Ren SX (2011) The genus *Chilocorellus* Miyatake, 1994 (Coleoptera: Coccinellidae) from Laos, with description of a new species. Pakistan journal of Zoology 43(1): 123–125.

[B17] Wang XM Huo LZ Ren SX (2014) Serangiini and Sticholotidini (Coleoptera: Coccinellidae) from Tibet, China. Annales Zoologici (Warszawa) 64(1): 15–31. 10.3161/000345414X680528

[B18] Wang XM Escalona HE Ren SX Chen XS (2017) Taxonomic review of the ladybird genus *Sticholotis* from China (Coleoptera: Coccinellidae). Zootaxa 4326(1): 001–072. 10.11646/zootaxa.4326.1.1

